# LyP‐1‐fMWNTs enhanced targeted delivery of MBD1siRNA to pancreatic cancer cells

**DOI:** 10.1111/jcmm.14864

**Published:** 2020-01-22

**Authors:** Quan‐Jun Lin, Zhi‐Bo Xie, Ya Gao, Yi‐Fan Zhang, Lie Yao, De‐Liang Fu

**Affiliations:** ^1^ Department of Pancreatic Surgery Pancreatic Disease Institute Huashan Hospital Shanghai Medical College Fudan University Shanghai China; ^2^ Department of General Surgery Tongren Hospital Shanghai Jiaotong University Medical College Shanghai China; ^3^ Department of Plastic & Reconstructive Surgery Shanghai Ninth People's Hospital School of Medicine Shanghai Jiao Tong University Shanghai China

**Keywords:** active targeting, LyP‐l peptide, multi‐walled carbon nanotubes, pancreatic cancer, RNA interference

## Abstract

Functionalized multi‐walled carbon nanotubes have been extensively gained popularity in pancreatic cancer gene therapy. LyP‐1, a peptide, has been proved to specifically bind pancreatic cancer cells. The potential therapeutic effect of LyP‐1–conjugated functionalized multi‐walled carbon nanotubes in treating pancreatic cancer is still unknown. In this study, LyP‐1–conjugated functionalized multi‐walled carbon nanotubes were successfully synthesized, characterized and showed satisfactory size distribution and zeta potential. Compared with functionalized multi‐walled carbon nanotubes, cellular uptake of LyP‐1–functionalized multi‐walled carbon nanotubes was shown to be increased. Compound of LyP‐1–functionalized multi‐walled carbon nanotubes and MBD1siRNA showed superior gene transfection efficiency. Moreover, LyP‐1‐fMWNTs/MBD1siRNA complex could significantly decrease the viability and proliferation and promoted apoptosis of pancreatic cancer cells in vitro. Further xenograft assays revealed that the tumour burden in the nude mice injected with LyP‐1–functionalized multi‐walled carbon nanotubes/MBD1siRNA was significantly relieved. The study demonstrated that LyP‐1–functionalized multi‐walled carbon nanotubes/MBD1siRNA could be a promising candidate for tumour active targeting therapy in pancreatic cancer.

## 1 INTRODUCTION

Pancreatic cancer is one of the most lethal malignant tumours in the world, with a 5‐year survival rate of <5%.^1^ Pancreatic ductal adenocarcinoma (PDAC) is the most common type of pancreatic cancer. Surgical resection offers the only radical chance for patients with PDAC; however, only 20% of patients are eligible for radical resection.^2^ Despite improvements in surgical techniques and adjuvant medical therapy, overall survival (OS) is still unsatisfied.^3^ It may accuse to the unclear mechanism of how PDAC develops and progresses.

The process of PDAC formation and development is complicated, among which DNA methylation, a well‐studied epigenetic modification, has been regarded as a key contributor to PDAC carcinogenesis.^4^ The hypermethylation of promoter of tumour suppressor genes was a characterized mechanism associated with PDAC carcinogenesis.^5^ The family of methyl‐CpG‐binding domain (MBDs) proteins have emerged as being associated with DNA methylation.^6^ As a transcriptional regulator, MBD1 could bind methylated CpG islands of tumour suppressor genes and represses their transcription to promote tumorigenesis.^6^ MBD1 has been reported to be overexpressed in PDAC tissues than normal pancreas, and the high expression level of MBD1 was associated with poor prognosis and lymphatic metastasis in patients with PDAC.^7^ Because of the importance of MBD1 in epigenetic regulation and transcriptional repression, it is a promising candidate as a gene therapeutic target for PDAC.

RNA interference (RNAi) is an effective strategy to silence genes and has shown great promise for anticancer therapy.^8^ The major advantages of RNAi over some small drug molecules are that the small interfering RNA sequences could be designed for highly specific inhibition of target of interest. Both viral and non‐viral delivery vectors were currently used in delivery of nucleic acids. Although viral delivery vectors had the advantages of achieving high levels of gene expression, the applications were limited for the immunogenic, induction of inflammation and the potential oncogenic effects.^9^ Non‐viral delivery vectors might be a more promising delivery systems as their safety concerns.

Our previous studies had shown that functionalized carbon nanotubes (fCNTs) could be used for targeted delivery of chemotherapeutic drug to PDAC and metastasis.^10^, ^11^ In addition, CNTs, as non‐viral vectors, were designed to be gene delivery carriers based on cationic polymers.^9^ Cationic CNTs were suggested to form stable complexes characterized by electron microscopy, surface plasmon resonance, electrophoresis and fluorescence dye exclusion.^9^ Several studies have reported the CNTs‐based siRNA delivery system showed efficacy in vitro and in vivo.^12^ Recently, the efficiency of DNA transfection using fCNTs delivery systems has been increased because of covalent modification of external walls of the tubes with polyethyleneimine (PEI).^13^ This modality prevents DNA from enzyme degradation and the enhanced proton sponge effects of PEI coating on the surface of CNTs. However, therapeutic efficacy was far from optimal resulting from poor tumour cell uptake, although a great deal of attempts had concentrated on gene delivery of fCNTs.^14^

The surface decoration of nanoparticles by a specific tumour homing ligand could increase the selective and efficient internalization by targeting tumour cells, which coined as an active targeting effect to tumour. Studies had shown that ligand‐directed active targeting nanoparticles present improved therapeutic performances comparing to their passive targeting counterparts.^15^ LyP‐1 (CGNKRTRGC) is a cyclic peptide binding to p32, a mitochondrial chaperone protein.^16^ In lymphatic endothelial cells, tumour cells and tumour macrophages, p32 is expressed at the cell surface, making it a tumour‐specific target.^17^ LyP‐1 has been used for active targeting of nanoparticles and drugs in breast cancer^18^ and lymph node metastasis.^19^ The aim of our study was to develop a gene delivery system of LyP‐1–conjugated functionalized multi‐walled carbon nanotubes (fMWNTs) combining active targeting and passive targeting to delivery plasmid DNA to PDAC.

## 2 MATERIALS AND METHODS

### 2.1 Ethics approval and consent to participate

The study was approved by the Institutional Review Board of Shanghai Medical College, Fudan University, China. This study was conducted in accordance with the Declaration of Helsinki. All animal experiments in this study were carried out according to the standards of animal care as outlined in the Shanghai Medical College of Fudan University.^20^

### 2.2 Synthesis and characterization of fMWNTs and LyP‐1‐fMWNTs

The preparation of oxidized MWNTs was through the combined treatment of strong acids and sonication which aimed to generate anionic groups (mainly carboxylate) along the sidewalls and ends of the nanotubes. For preparation of fMWNTs (MWNTs‐PEG‐PEI conjugated), an oxidized MWNT solution (1 mg/mL) was mixed with maleimide‐PEG‐NH_2_ and methoxy PEG‐NH_2_ (1 mg/mL, mass ratio, 1:4), added with EDC (1 mg/mL).

The synthesis of LyP‐1‐fMWNTs was functionalized by PEI and processed the reaction between LyP‐1 with sulfhydryl group and the maleimide functional group located at the distal end of PEG surrounding the fMWNTs. LyP‐1 was mixed with fMWNTs at a peptide:maleimide molar ratio of 1.3:1. The conjugation of LyP‐1 to maleimide on the fMWNTs was performed for 2 hours at a low speed rotation in protection of nitrogen away from light. Cyclization of conjugated LyP‐1 was performed by Acm deprotection at Cys2 and Cys10 using iodine reagent. Iodic methanol solution was immediately added at a peptide:iodine molar ratio of 2:1 and stirred under protection of HAc at room temperature for 4 hours.

**Figure 1:**
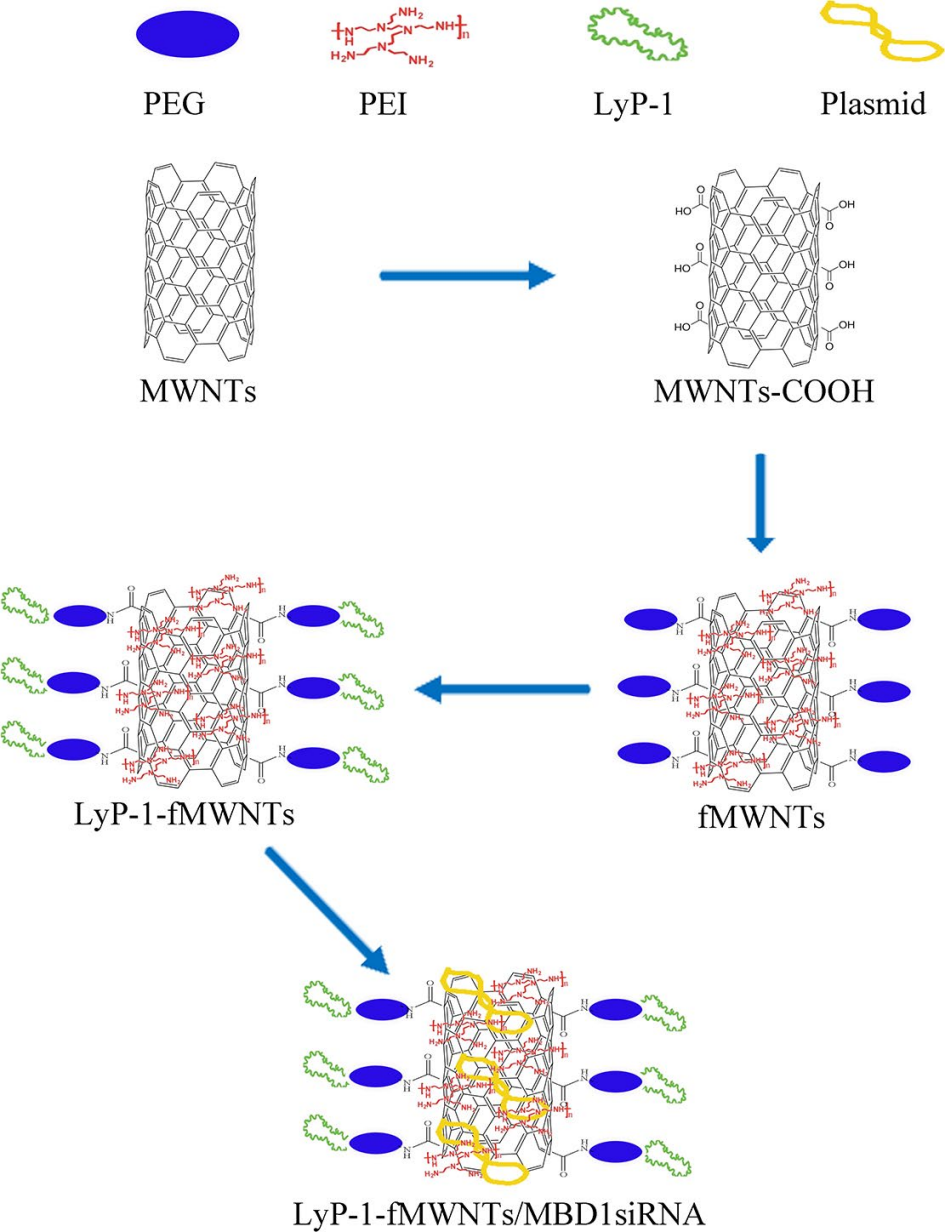
The schematics of the synthetic strategy of LyP‐1‐fMWNTs

The mean diameter and zeta potential of the fMWNTs and LyP‐1‐fMWNTs were determined by dynamic light scattering method using affordable molecular/ particle size and zeta potential analyzer (Malvern Zetasizer Nano, UK). The morphology of fMWNTs and LyP‐1‐fMWNTs was observed by transmission electron microscope (TEM, JEM‐200CX, Japan). The reaction solution was dialysed against distilled water to remove salts and lyophilized to obtain fMWNTs and LyP‐1‐fMWNTs, which was characterized by Fourier‐transform infrared (FTIR) spectra on a AVATAR 360 spectrometer (Nicolet). LyP‐1‐fMWNTs and fMWNTs (synthesized by maleimide‐PEG‐NH_2_) were dissolved in deuteroxide in a glass NMR tube and analysed on a Bruker Avance 500 AV system (Germany) using standard proton NMR to verify LyP‐1 with sulfhydryl group conjugation to maleimide functional group. Elemental analysis data of fMWNTs and LyP‐1‐fMWNTs were obtained by an Elemental Analyzer (VARIO ELIII, Elementar). The schematics of the synthetic strategy of LyP‐1‐fMWNTs were showed in Figure 1.

### 2.3 Cell lines and cell culture

Cell lines (HPDE6‐C7, SW1990, BxPC‐3) were obtained from the Type Culture Collection of the Chinese Academy of Sciences (Shanghai, China) and were used in this study. Cells were routinely cultivated in RPMI 1640 culture medium and supplemented with 10% FBS, 100 U/mL penicillin and 100 U/mL streptomycin, at 37°C in 5% CO_2_ and 95% air atmosphere and >95% humidity.

### 2.4 Cell Counting Kit‐8 assays

The various concentrations of fMWNTs and LyP‐1‐fMWNTs resuspended in 200 μL RPMI‐1640 medium were added to the cells, which were incubated for 24 hours at 37°C. After incubation, CCK‐8 reagent (KeyGen BioTech) was added to the cells and cultured. The absorbance was measured at 450 nm using a microplate reader (Tecan Safire2, Switzerland).

### 2.5 EdU assays

We used Click‐iT EdU Alexa Fluor 488 Imaging Kit (Invitrogen) in this study for detecting the proliferation of PDAC cell line according to the manufacturer's instructions. Fluorescence was analysed using a Zeiss 510 laser scanning microscope (Zeiss).^20^

### 2.6 Cellular uptake of fMWNTs and LyP‐1‐fMWNTs

For microscopic observation, cells were added with 3 μg/mL fMWNTs‐FITC and LyP‐1‐fMWNTs‐FITC. After incubation, the cells were washed with PBS, fixed with 4% formaldehyde, stained with DAPI and imaged by a fluorescence microscope (Micropublisher 3.3RTV, Olympus). To study the intracellular localization, cells were incubated with LyP‐1‐fMWNTs‐FITC. After that, cells were cultured with fresh medium containing 50 nmol/L Lyso‐Tracker Red (Invitrogen). Afterwards, cells were washed with PBS, fixed with 4% formaldehyde, stained with DAPI and visualized by a laser scanning confocal microscopy (LSM 710, Zeiss).

### 2.7 Agarose gel electrophoresis

LyP‐1‐fMWNTs/MBD1siRNA was prepared by mixing 0.2 μg of pDNA with different concentration LyP‐1‐fMWNTs solutions to obtain compounds with various N/P ratio (7:1, 14:1, 28:1, 42:1, 56:1, 70:1 and 84:1). Compounds were firstly incubated and loaded into parallel wells of a 0.7% agarose gel with 0.5 μg/mL ethidium bromide. Electrophoresis was run at a voltage of 100 mV for 90 minutes and using 0.5 X TBE as a running buffer. The bands of pDNA were visualized by ethidium bromide staining and imaged by UV light using the Fluor Chem Imaging System (Alpha Innotech).

### 2.8 Gene transfection and the GFP expression

Briefly, 2 μg pDNA plasmid and designated concentration of fMWNTs or LyP‐1‐fMWNTs were diluted in 100 μL OptiMEM medium, respectively. The diluted fMWNTs or LyP‐1‐fMWNTs were added to the pDNA solution. After being pipetted mixed, the mixtures were added with cells. Finally, the GFP‐expressed cells that emitted fluorescence were imaged by a fluorescence microscope (Micropublisher 3.3RTV, Olympus, Japan) and the transfection efficiency was analysed by flow cytometer (BD Calibur, USA) after additional 48 hours incubation at 37°C.

### 2.9 Cell apoptosis assay

The cell apoptosis rate was assessed using the PE Annexin V Apoptosis Detection Kit I (BD Pharmingen, USA) according to the instructions. The cells were grouped and treated as described for gene transfection. The cells in each group were collected and resuspended in 1X binding buffers. Then, 5 µL of PE Annexin V and 5 µL of 7‐AAD solution were added. The stained cells were gently vortexed and incubated. Each tube was added 400 µL of 1X Binding Buffer. Finally, the suspension was subjected to the flow cytometry analysis (BD Calibur, USA).

### 2.10 Real‐time quantitative PCR

Total RNA was isolated from the PDAC cells using TRIzol reagent (Invitrogen). The RNA was reversely transcribed into cDNA with Oligo (dT) and M‐MLV Reverse Transcriptase (Thermo Fisher Scientific). GAPDH was used as a reference gene.

### 2.11 Western blotting

The total cell lysate was separated on a 10% sodium dodecyl sulphate‐polyacrylamide gel using electrophoresis (SDS‐PAGE) and transferred onto a PVDF membranes. The membrane was blocked for 1 hour at room temperature in 10% FBS and then incubated overnight at 4°C with different primary antibodies. The primary antibodies were as follows: anti‐MBD1, 1:1000; GAPDH, 1:10 000 (Abcam). After washing, the membrane was incubated with secondary antibody. The signal was detected by the ECL detection system (Chemicon).

### 2.12 Xenograft mouse model

BALB/c nude mice (4‐5 weeks old, 18‐20 g) used in this study were obtained from Shanghai Medical College of Fudan University. All mice were bred in a specific pathogen‐free (SPF) laboratory in the animal centre of Shanghai Medical College of Fudan University. All nude mice were injected 1 × 10^7^ cells suspension into the right upper flanks. Four weeks after tumour inoculation, all nude mice were divided into 4 groups (control group, n = 5; MBD1siRNA group, n = 5; fMWNTs/MBD1siRNA group, n = 5; and LyP‐1‐fMWNTs/MBD1siRNA group, n = 5). Tumour volume = (width^2^ × length)/2. At 15th day post‐initial administration, all nude mice were killed and subcutaneous tumours were removed.

### 2.13 Statistical analysis

Measurement variable was presented as mean ± SD. Significant differences between two groups were determined using Student's *t* test, whereas multiple groups were analysed by one‐way analysis of variance with Fisher's least significant difference. All the statistical analyses were performed with SPSS 22.0 software package. *P* < .05 was considered to indicate a statistically significant difference.

## 3 RESULT

### 3.1 Characterization of fMWNTs and LyP‐1‐fMWNTs

**Figure 2:**
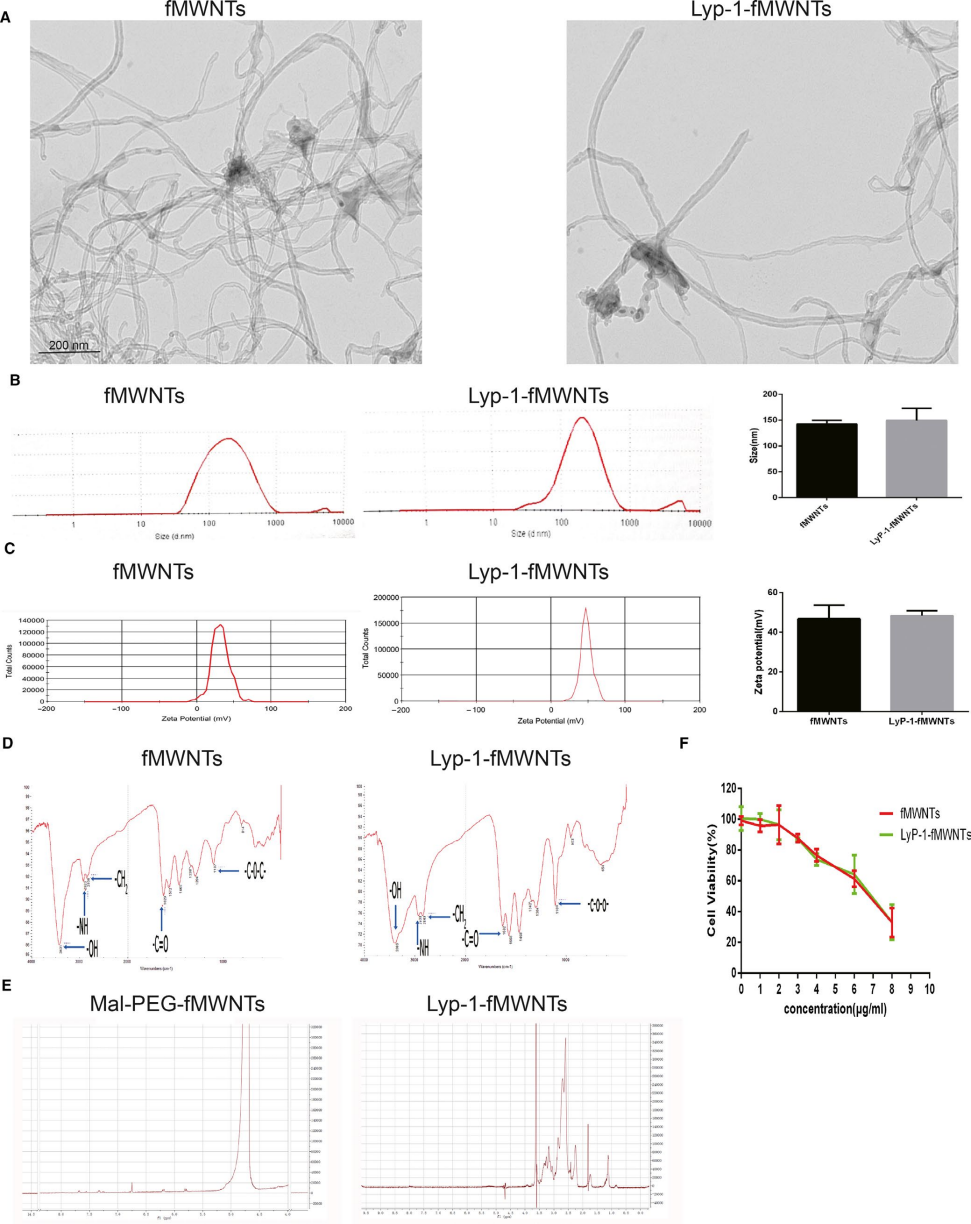
Characterization of fMWNTs and LyP‐1‐fMWNTs. A, TEM images of fMWNTs and LyP‐1‐fMWNTs. B, The particle size and size distribution of fMWNTs and LyP‐1‐fMWNTs. C, The zeta potential of fMWNTs and LyP‐1‐fMWNTs. D, The FTIR spectra of fMWNTs and LyP‐1‐fMWNTs. E. The NMR spectra of fMWNTs and LyP‐1‐fMWNTs. F. The cytotoxicity of fMWNTs and LyP‐1‐fMWNTs in the PDAC cell

As shown in the TEM images (Figure 2A), both the length of fMWNTs and LyP‐1‐fMWNTs were less than 200 nm. There were some lumps onto the surface of MWNTs' tubular micromorphology, which indicated the carboxylic acid groups, PEG and PEI were adhered to the nanotubes. From the data of particle size (Figure 2B‐D), no significant difference was observed between fMWNTs (142.4 ± 7.15 nm) and LyP‐1‐fMWNTs (149.6 ± 23.62 nm). The zeta potential analysis indicated that the fMWNTs (46.76 ± 7.05 mV) and LyP‐1‐fMWNTs (48.37 ± 2.58 mV) both bear a positive charge, which resulting from the functionalized PEI onto the surface of MWNTs. Elemental analysis showed that nitrogen content was 17.68% and 18.45% for PEI in fMWNTs and LyP‐1‐fMWNTs, respectively. The molar ratio of carbon nanotube and PEI was 1:1.83 in both fMWNTs and LyP‐1‐fMWNTs.

The functional groups of fMWNTs and LyP‐1‐fMWNTs were characterized by FTIR. It could be observed the absorption peak at 3431 cm^−1^, 2923 cm^−1,^ 2856 cm^−1^, 1629 cm^−1^ and 1107 cm^−1^, being assigned to the stretching vibration of ‐OH, ‐N‐H‐, ‐CH_2_, ‐C=O and ‐C‐O‐C‐ for the FTIR spectra of fMWNTs, whereas the FTIR spectrum of LyP‐1‐fMWNTs exhibited the absorption peak at 3389 cm^−1^, 2930 cm^−1^, 2849 cm^−1^, 1632 cm^−1^ and 1109 cm^−1^ also being assigned to the stretching vibration of ‐OH, ‐N‐H‐, ‐CH_2_, ‐C=O and ‐C‐O‐C‐ (Figure 2D). These peaks were typical characteristics of PEI and PEG, which indicated the functional carboxyl group and the conjugation of PEI and PEG. In the spectrum of fMWNTs (synthesized by maleimide‐PEG‐NH2), the maleimide‐PEG‐fWMNTs have the characteristic peak at 6.7 ppm (Figure 2E). However, the characteristic peak was invisible in the proton NMR spectrum of LyP‐1‐fMWNTs, which indicated that the thiol group of LyP‐1 had reacted with the MAL group.

### 3.2 Cytotoxicity of fMWNTs and LyP‐1‐fMWNTs

The cellular viability of BxPC‐3 cells decreased gradually with the increase in fMWNTs or LyP‐1‐fMWNTs concentration (Figure 2F). Comparing the cytotoxicity between fMWNTs and LyP‐1‐fMWNTs, there was no significant difference with the same concentration. From the results, the cellular viability decreased significantly after incubating with the concentration over 3 μg/mL. The viability could be above nearly 85% when the concentration below 3 μg/mL.

### 3.3 Cellular uptake of fMWNTs and LyP‐1‐fMWNTs

**Figure 3:**
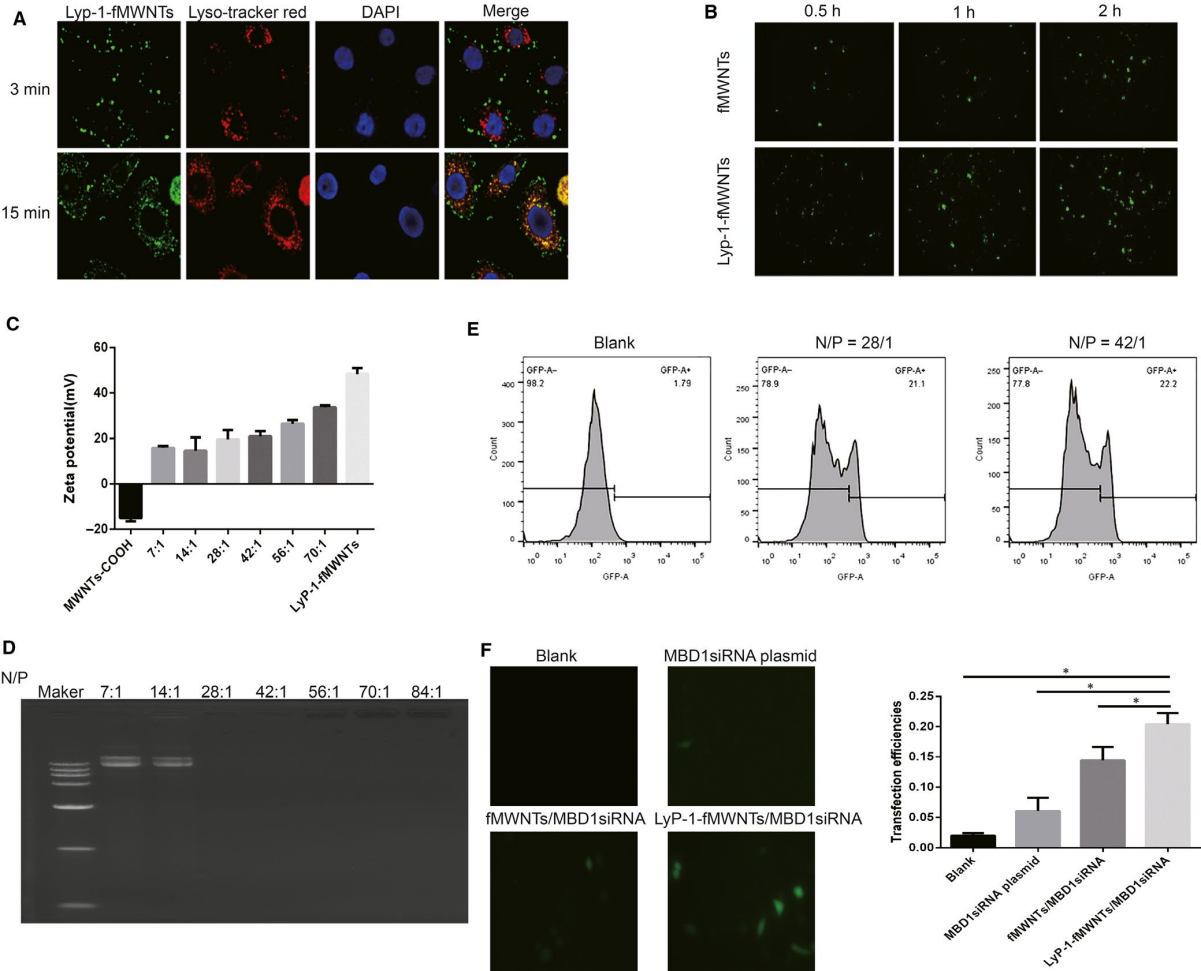
LyP‐1–modified fMWNTs enhanced the targeting for pancreatic cancer cells. A, Confocal images of BxPC‐3 cells incubated with LyP‐1‐fMWNTs (400×). B, Cellular uptake of BxPC‐3 cells incubated with fMWNTs‐FITC and LyP‐1‐fMWNTs‐FITC (400×). C, The zeta potential of MWNT‐COOH and LyP‐1‐fMWNT/MBD1 siRNA with N/P ratio of 7, 14, 28, 42, 56, 70. D, Agarose gel electrophoresis of pDNA released from LyP‐1‐fMWNT/MBD1 siRNA with N/P ratio of 7, 14, 28, 42, 56, 70 and 84. E, The transfection efficiencies of LyP‐1‐fMWNT/MBD1 siRNA compound with N/P ratio of 28 and 42. F, Fluorescence images of 4 different MBD1 siRNA‐transfected BxPC‐3 cells (200×)

We found that fMWNTs‐FITC and LyP‐1‐fMWNTs‐FITC could be internalized by PDAC cells (Figure 3A). At the same time‐points, FITC‐positive cells in LyP‐1‐fMWNTs‐FITC incubated group were more than that in fMWNTs‐FITC incubated group (Figure 3B). It demonstrated that there was an active targeting role of LyP‐1 in LyP‐1‐fMWNTs uptake by BxPC‐3 cells in vitro. We also tested the cellular uptake of fMWNTs and LyP‐1‐fMWNTs in human normal pancreatic ductal epithelium cell (HPDE6‐C7). We found that fMWNTs‐FITC and LyP‐1‐fMWNTs‐FITC could be also internalized by HPDE6‐C7 cell and FITC‐positive cells in LyP‐1‐fMWNTs‐FITC group were similar with that in fMWNTs‐FITC incubated group (Figure S1). However, FITC‐positive PDAC cells in LyP‐1‐fMWNTs‐FITC incubated group were significantly more than that in fMWNTs‐FITC incubated group (Figure 3B). The results indicated that LyP‐1‐fMWNTs is more efficiently internalized into cells than fMWNTs because of the specificity of Lyp‐1 to PDAC cells.

As shown in Figure 3A, fluorescence confocal localization analysis found that LyP‐1‐fMWNTs‐FITC in cells had a high coincidence of lysosomes labelled by Lyso‐Tracker Red. These results suggested that LyP‐1‐fMWNTs mainly located inside cell endosomes and lysomes after entering cells, which were the further evidence to indicate the uptake of LyP‐1‐fMWNTs by BxPC‐3 cells and an uptake pathway via endocytosis.

### 3.4 Characterization of LyP‐1‐fMWNTs/MBD1siRNA compounds

The zeta potentials of MWNTs‐COOH, LyP‐1‐fMWNTs/MBD1siRNA with the N/P ratio of 7, 14, 28, 42, 56, 70 and LyP‐1‐fMWNTs were 14.93 ± 1.51 mV, 15.87 ± 0.86 mV, 14.63 ± 5.86 mV, 19.63 ± 4.05 mV, 20.90 ± 2.33 mV, 26.53 ± 1.59 mV, 33.80 ± 0.72 mV and 48.37 ± 2.58 mV, respectively, with a gradual increasing trend (Figure 3C).

Agarose gel retardation assay demonstrated the ability of LyP‐1‐fMWNTs to bind pDNA, as only free pDNA was able to migrate in the gel. Upon complexation of pDNA with the nanotubes, the fluorescent signal was either quenched or retained in the wells depending on the degree of pDNA condensation. The significant retardation of LyP‐1‐fMWNTs/MBD1siRNA compounds was observed when LyP‐1‐fMWNTs/MBD1siRNA compounds were at high N/P ratios. As shown in Figure 3D, complete complexation with pDNA of LyP‐1‐fMWNTs was observed at an N/P ratio of 28. While this condition was enough to completely retard pDNA moving in electrophoresis assay, indicating all pDNA was bound to the compounds in this condition (at an N/P ratio of 28). These results suggested that a higher N/P ratio of 28 was required for complete loading of pDNA on LyP‐1‐fMWNTs/MBD1siRNA compounds.

### 3.5 Enhanced gene transfection efficiency with LyP‐1 modified

To choose the optimal N/P ratio for gene transfection to BxPC‐3 cells, the transfection ability of N/P ratio of 28 and 42 was evaluated for comparison. The transfection efficiencies of N/P ratio of 28 and 42 were 21.1% and 22.2%, indicating there was no significant difference between the two groups. In consideration of the lower cytotoxicity, N/P ratio of 28 was selected to apply to the transfection experiments (Figure 3E).

As shown in the Figure 3F, all three transfected groups could deliver GFP gene into BxPC‐3 cells. However, three transfected groups exhibited different transfection efficiencies. At N/P ratio of 28, the transfection efficiencies of MBD1siRNA plasmid groups, fMWNTs/MBD1siRNA groups and LyP‐1‐fMWNTs/MBD1siRNA groups were 6.09 ± 2.21%, 14.43 ± 2.25% and 20.40 ± 1.85%, indicating the two carbon nanotubes transfected groups had better transfection capacity than MBD1siRNA plasmid groups. We found that naked MBD1siRNA plasmid groups failed to render appreciable GFP expression to the treated BxPC‐3 cells. And the higher GFP expression was observed among two carbon nanotube transfected groups. The similar results could obtain from the transfection efficiencies analysing through the flow cytometry (Figure 3F). Among two carbon nanotubes transfected groups, LyP‐1 modification carbon nanotubes transfected groups had the significant higher transfection efficiencies comparing with fMWNTs/MBD1siRNA‐treated cells.

### 3.6 MBD1 gene expression in pancreatic cancer cells after RNA interference of carbon nanotube/MBD1siRNA compounds

**Figure 4:**
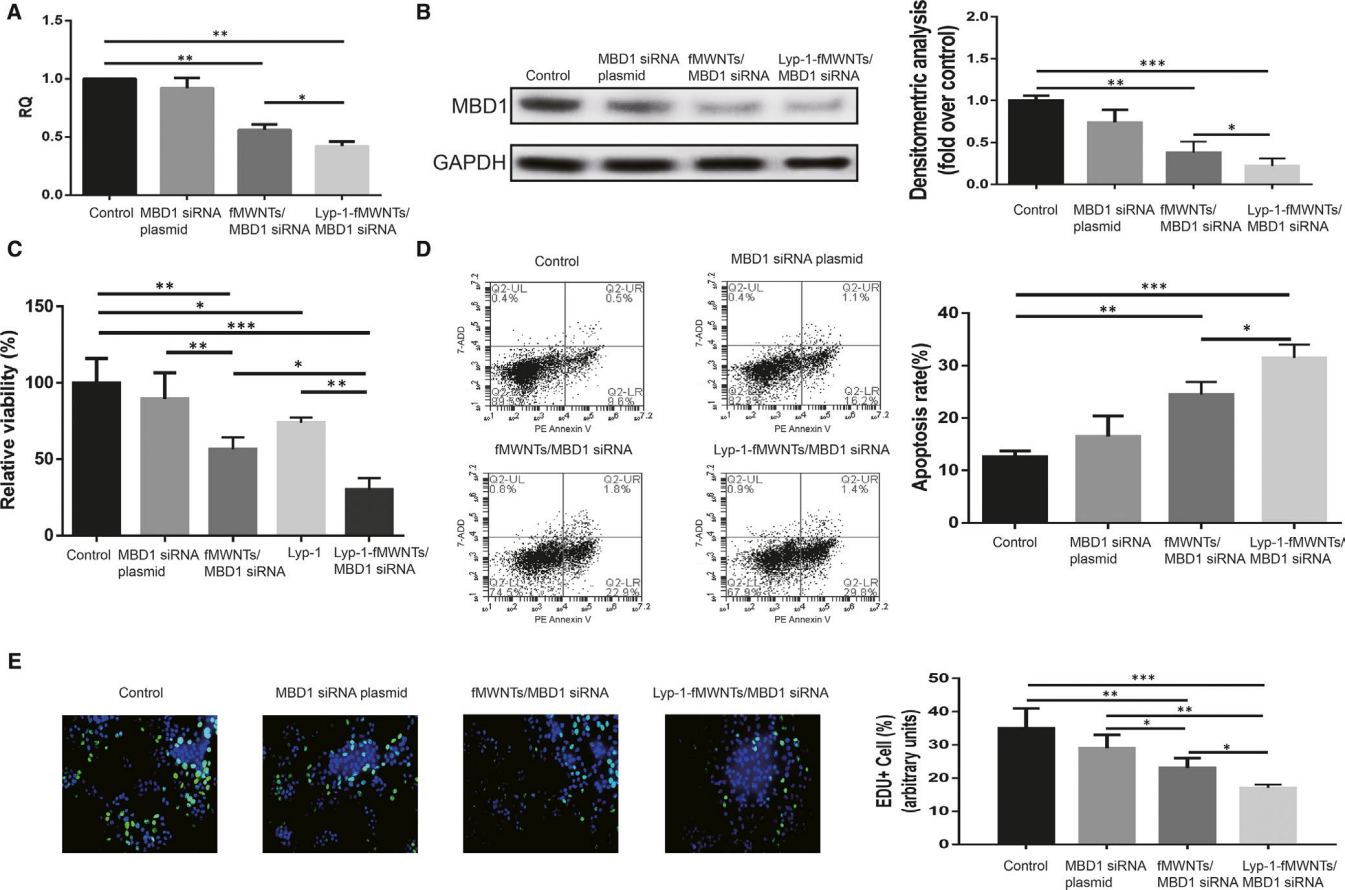
LyP‐1‐fMWNTs/MBD1siRNA inhibits PDAC cell viability and proliferation, and promotes PDAC cell apoptosis. A, The relative quantitation of MBD1 in BxPC‐3 cells after RNA interference. B, The expression of MBD1 protein in BxPC‐3 cells after RNA interference. C, The cell viability of BxPC‐3 cells after RNA interference. D, PE Annexin V/7‐AAD cytometry plots of BxPC‐3 cells after RNA interference. E, EdU‐positive BxPC‐3 cells after RNA interference

Our study found that MBD1 gene expression was decreased after RNA interference of MBD1 gene in PDAC cells among these transfected groups, especially in the two carbon nanotubes transfected groups (Figure 4A, and Figure S2A). LyP‐1‐fMWNTs/MBD1siRNA groups showed a strong RNA interference effect with a decrease in MBD1 mRNA level comparing to the groups without LyP‐1 modification. Afterwards, LyP‐1 modification carbon nanotube significantly enhanced down‐regulation of MBD1 protein expression in LyP‐1‐fMWNTs/MBD1siRNA‐treated cells, consistent with the qRT‐PCR results (Figure 4B, and Figure S2B).

### 3.7 LyP‐1‐fMWNTs/MBD1siRNA inhibits PDAC cell viability and proliferation, and promotes PDAC cell apoptosis

Cell viabilities were significantly decreased in carbon nanotubes groups. Among the two carbon nanotubes groups, the cell viability in LyP‐1‐fMWNTs/MBD1siRNA groups was significantly lower comparing to that in fMWNTs/MBD1siRNA groups. In order to clarify the intrinsic cytotoxicity of Lyp‐1 peptide, we tested the cell viability of Lyp‐1 peptide alone and found the cell viability with Lyp‐1 alone is lower than PDAC cells in control groups. Nevertheless, the cell viability in LyP‐1‐fMWNTs/MBD1siRNA groups is significantly lower than that in Lyp‐1 group. The results confirmed that the LyP‐1‐fMWNTs/MBD1siRNA could effectively inhibite PDAC cell viability because of the specificity of Lyp‐1 targeting delivery of MBD1siRNA to PDAC cells besides Lyp‐1 its intrinsic cytotoxicity (Figure 4C and Figure S2C). Both fMWNTs/MBD1siRNA groups and LyP‐1‐fMWNTs/MBD1siRNA groups had a higher apoptosis rates than the other two groups (*P* < .05) (Figure 4D and Figure S2D). And among the two groups, apoptosis rates in LyP‐1‐fMWNTs/MBD1siRNA groups were higher than those in fMWNTs/MBD1siRNA groups, which indicated the effect of inducing apoptosis was significantly enhanced (*P* < .05) with LyP‐1 modification. Our EdU incorporation assays demonstrated that the proportion of EdU‐positive cells in fMWNTs/MBD1siRNA and LyP‐1‐fMWNTs/MBD1siRNA group decreased markedly than other 2 groups (Figure 4E and Figure S2E). However, EdU‐positive cells were significantly less in LyP‐1‐fMWNTs/MBD1siRNA group than fMWNTs/MBD1siRNA group.

### 3.8 LyP‐1‐fMWNTs/MBD1siRNA inhibits PDAC growth in vivo

**Figure 5:**
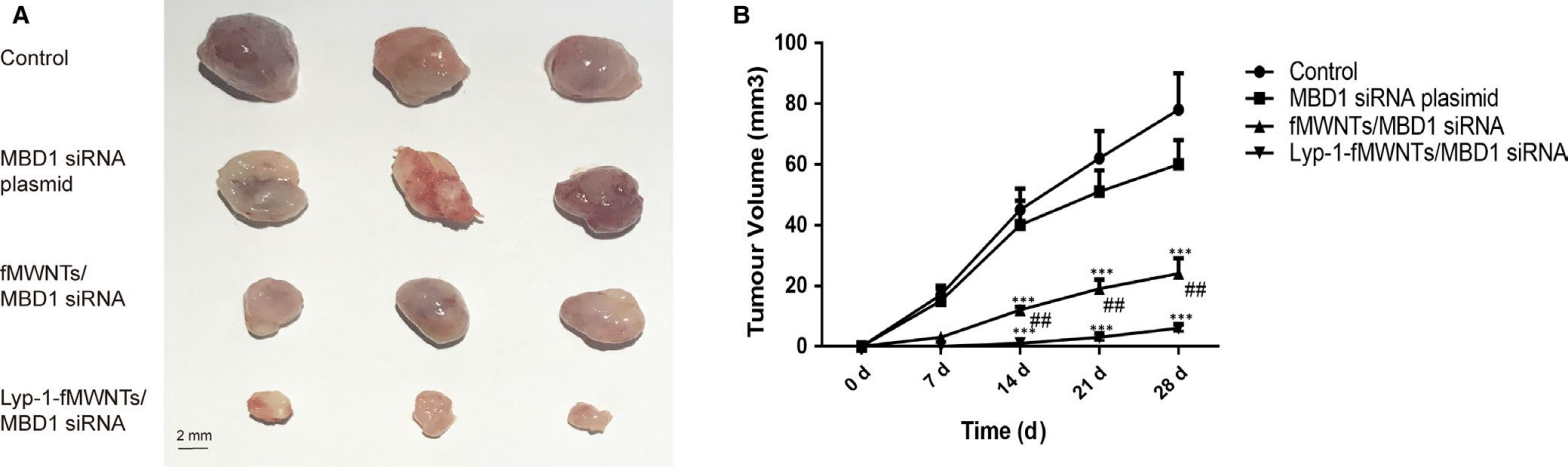
LyP‐1‐fMWNTs/MBD1siRNA inhibits PDAC growth in vivo. A, Images of tumours in xenograft mouse models. B, Tumour volume was measured using a caliper at indicated time‐points

Xenograft assays revealed that the tumour burden in the nude mice injected with fMWNTs/MBD1siRNA and LyP‐1‐fMWNTs/MBD1siRNA was relieved than that in the nude mice injected with saline and MBD1siRNA. Compared with mice in fMWNTs/MBD1siRNA group, the tumour burden of mice in Lyp‐1‐fMWNTs/MBD1siRNA was significantly lighter (Figure 5).

## 4 DISCUSSION

Nowadays, the use of a new carrier, fMWNTs, is growing more and more popular in target therapy of PDAC. We have previously proved that fMWNTs could be used for targeted delivery of chemotherapeutic drug to PDAC and metastasis.^10^, ^11^, ^21^ In this study, we have designed and prepared a new carrier called Lyp‐1‐fMWNTs complex for target gene therapy in PDAC. We found that Lyp‐1‐fMWNTs could target PDAC cells and LyP‐1‐fMWNTs/MBD1siRNA complex could inhibit PDAC cell malignant ability both in vitro and in vivo.

Currently, more and more studies have proved that fMWNTs are gradually applied in the field of tumour therapy, vaccine rendering system besides optical use.^22^ The solubility and cytotoxicity are the major concerns for the use of fMWNTs. The cytotoxicity may be dependent on some aspects including physical properties and surface functionalization. Some groups had successfully used well functionalized and serum stable CNTs for experiments without observing apparent toxicity.^23^ Both in vitro and in vivo studies revealed that PEG‐modified CNTs had favourable pharmacokinetic and toxicology profiles. PEG‐modified CNTs had been successfully tested in pre‐clinical studies.^24^ In our study, PEG‐PEI–modified fMWNTs were synthesized, and it has a high dispersion and stability in water. The cytotoxicity of fMWNTs was both dose‐dependence showed the comfortable cell toxicity at the concentration below 3 μg/mL.

Cell surface p32, the target of LyP‐1 homing peptide, was up‐regulated in tumour endothelial cells (blood and lymphatic) and tumour cells.^25^ p32 has been regarded as a tumour‐specific target, and LyP‐1 had been used for targeting of drugs and nanoparticles in tumour and metastatic tumours in lymph nodes.^26^ The application of LyP‐1 peptides as coating surface to enhance drug delivery had been recently investigated.^19^ In this study, we used LyP‐1‐fMWNTs as a gene delivery system. We first tested the effect of LyP‐1 modification on cytotoxicity and found LyP‐1 peptide did not seem to affect the cytotoxicity. Next cellular uptake analysis results showed that there was an active targeting role of LyP‐1 in LyP‐1‐fMWNTs uptake by BxPC‐3 cells. fMWNTs were one of the best candidates for delivery vehicles among a wide range of different cell types. Currently, there were three different mechanisms for the cellular internalization of fMWNTs: internalization by endocytosis, internalization by phagocytosis and direct translocation through the plasma membrane.^27^, ^28^ Endocytosis described the internalization of macromolecules by cells through the formation of a vesicle routing to endosomes and lysosomes. Phagocytosis was similar to the endocytosis in principle.^29^ The difference between them was that the particles were considerably larger and the cell type was a professional phagocytic cell. The direct translocation through cell membrane suggested a mechanism similar to the nanoneedles, perforating and diffusing through the lipid bilayer of plasma membrane. We further investigated the mechanism of uptake of LyP‐1‐fMWNTs and found LyP‐1‐fMWNTs mainly located inside cell endosomes and lysomes after entering cells, which were the further evidence to indicate the uptake of LyP‐1‐fMWNTs by BxPC‐3 cells and an uptake pathway via endocytosis.

RNAi is an effective strategy to silence genes and has shown great promise for anticancer therapy.^30^ The application of viral delivery vectors was limited for the immunogenic, because of the possibility of giving rise to inflammation and the potential oncogenic effects.^9^ fMWNTs, as the new genetic carrier, could both connect siRNA and target specific tumour cells. MBD1, which was a transcriptional regulator that could repress the tumour suppressor genes through binding their methylated CpG islands, was strongly suggested to promote tumorigenesis.^6^ In PDAC, silencing MBD1 expression using RNAi would dramatically inhibit cell growth and induce cell apoptosis.^7^ More detailed studied also revealed that the MBD1 could affect the invasion, metastasis sensitivity of cells to radiation and chemical therapy in PDAC.^31^, ^32^ Our study incorporated the MBD1siRNA plasmid into the carbon nanotube carriers to confirm the therapeutic effect in PDAC. However, the similar MBD1 nanoparticles had no specificity to PDAC cells. In our research, we combined the enhanced permeability and retention (EPR)‐mediated passive targeting and specific ligand‐mediated active targeting to enhance the therapeutic effect in PDAC. On the one hand, the RNAi was combined with the carbon nanotubes to achieve passive tumour targeting by virtue of EPR effect in cancer, and on the other hand, we further modified the surface of functionalized CNTs with LyP‐1 to attain active tumour targeting in PDAC, which resulted from the affinity ability of LyP‐1 peptides with BxPC‐3 cells. The results showed that LyP‐1‐fMWNTs may be a potential gene carrier combined passive and active targeting for PDAC, and LyP‐1‐fMWNTs/MBD1siRNA produced significantly improved antitumour efficacy compared with the control groups both in vivo and in vitro.

## 5 CONCLUSION

CNTs were functionalized by PEG and PEI polymers and modified by LyP‐1 peptides for pDNA and MBD1siRNA delivery with improvement in gene transfection and antitumour efficacy because of the combination of passive target and active targeting. LyP‐1‐fMWNTs could delivery MBD1siRNA to PDAC cells more efficiently, and it has the potential to be a promising gene carrier in PDAC.

## CONFLICT OF INTEREST

The authors declare no competing interests in this work.

## AUTHOR CONTRIBUTIONS

Quanjun Lin and Zhibo Xie conceived and designed the experiments; Quanjun Lin and Ya Gao performed the experiments and drafted the manuscript; Quanjun Lin, Zhibo Xie and Ya Gao made products characterization and partially performed the experiments; Deliang Fu and Lie Yao edited and revised the manuscript; Yifan Zhang, Lie Yao and Deliang Fu advised the work and edited the manuscript; Yifan Zhang, Lie Yao and Deliang Fu contributed to the conception and design of the experiments and final approval of it. All authors have reviewed and approved the content of the submitted manuscript.

## Data Availability

The data that support the findings of this study are available from the corresponding author upon reasonable request.
