# Supplementary figures and images for: LyP‐1‐fMWNTs enhanced targeted delivery of MBD1siRNA to pancreatic cancer cells
Source: J Cell Mol Med. 2020 Jan 22;24(5):2891–900. doi: 10.1111/jcmm.14864 (PMC7077559; doi:10.1111/jcmm.14864)

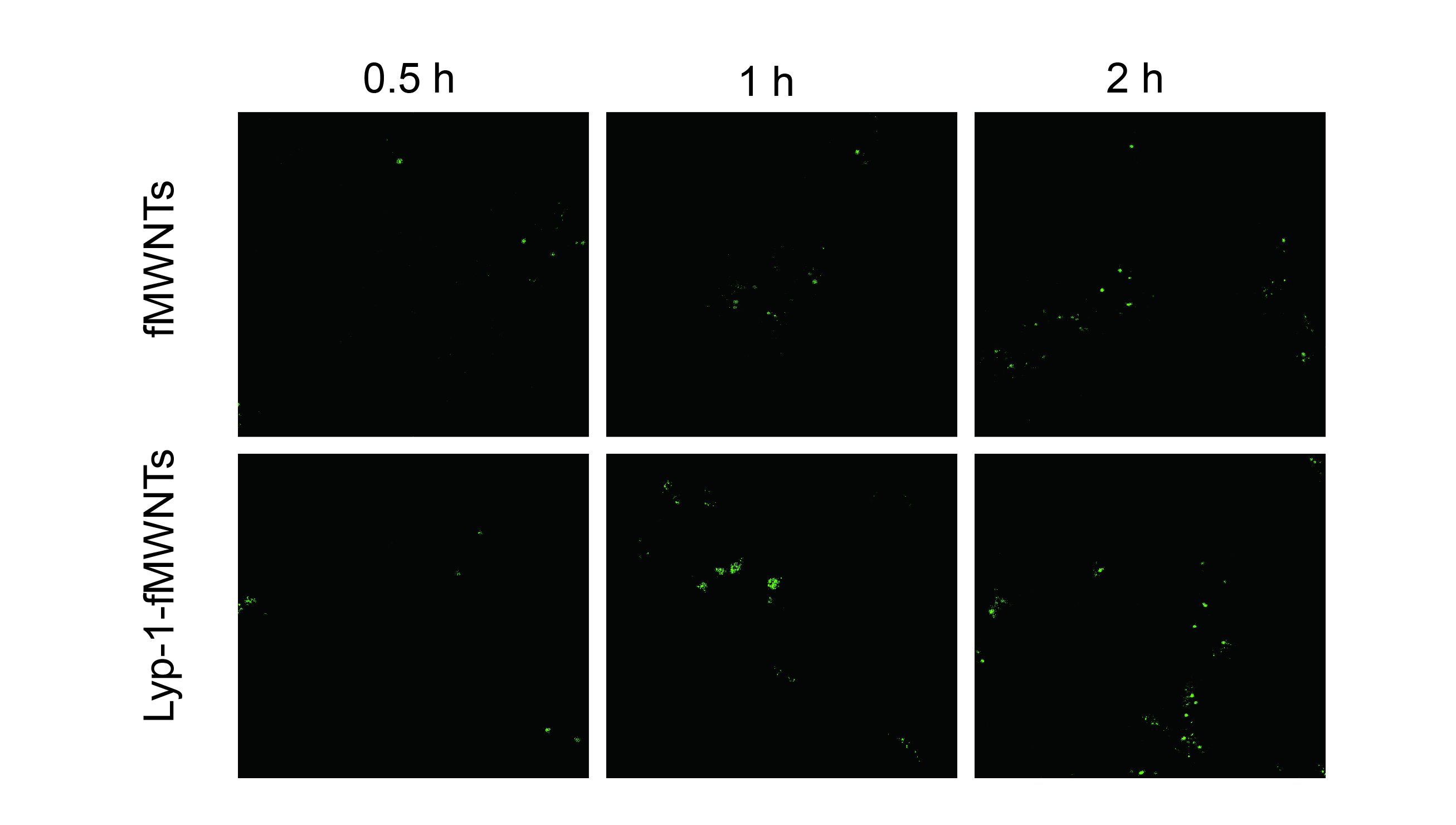

Supplement: Supplementary file 1 [file JCMM-24-2891-s001.tif]

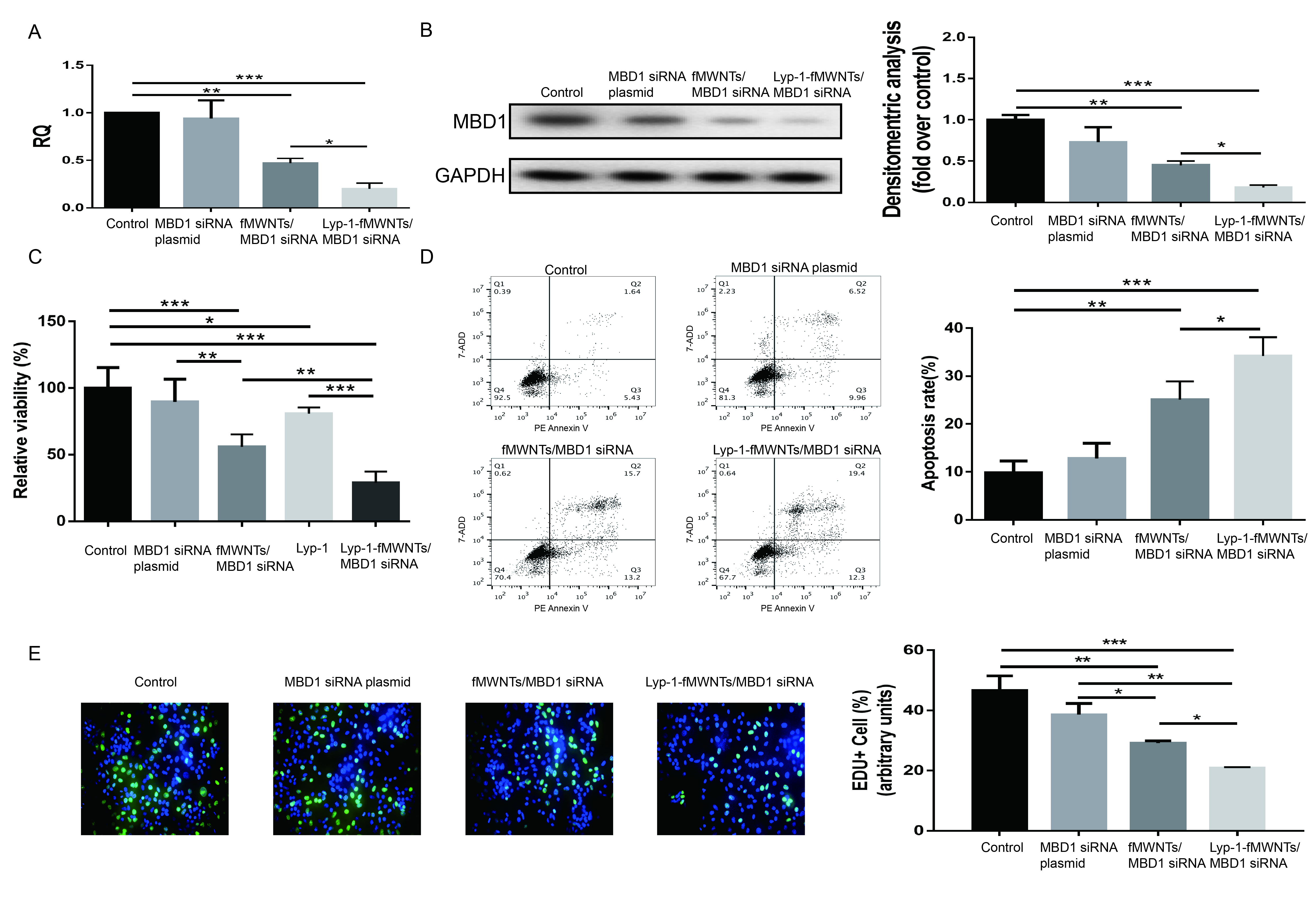

Supplement: Supplementary file 2 [file JCMM-24-2891-s002.tif]
